# RESIST ACINETO test for the rapid detection of NDM and OXA acquired carbapenemases directly from blood culture in *Acinetobacter* species

**DOI:** 10.1128/spectrum.01044-24

**Published:** 2024-08-20

**Authors:** Anaïs Potron, Marion Daniel, Mila Bay, Pauline Choulet, Thomas Garrigos, Loïk Sababadichetty, Olivier Belmonte, Damien Fournier, Katy Jeannot, Guillaume Miltgen

**Affiliations:** 1 Laboratoire Associé au Centre National de Référence de la Résistance aux Antibiotiques, Centre Hospitalier Universitaire de Besançon, Besançon, France; 2 Université de Franche-Comté, CNRS, UMR 6249 Chrono-environnement, Besançon, France; 3 UMR Processus Infectieux en Milieu Insulaire Tropical, CNRS 9192, INSERM U1187, IRD 249, Université de La Réunion, Saint-Denis, La Réunion, France; 4 Laboratoire de Bactériologie, Centre Hospitalier Universitaire de La Réunion, site Félix Guyon, Saint-Denis, La Réunion, France; 5 Centre Régional en Antibiothérapie de La Réunion (CRAtb Réunion), Saint-Pierre, La Réunion, France; University Paris-Saclay, Clamart, France

**Keywords:** *Acinetobacter*, carbapenemases, OXA-23, OXA-24/40, OXA-58, NDM, rapid detection, blood culture

## Abstract

Carbapenem-resistant *Acinetobacter baumannii* (CRAB) are increasingly reported worldwide and a leading cause of mortality associated with antimicrobial resistance. Their early detection, particularly in the cases of bloodstream infections, is crucial in attempting to initiate effective antibiotic treatment. The immunochromatographic assay RESIST ACINETO (Coris BioConcept) is a new test developed for the detection of OXA-23, OXA-40/58, and New-Delhi Metallo-beta-lactamase (NDM) carbapenemases in *Acinetobacter* spp. We evaluated this test on a collection of 121 *Acinetobacter* spp. clinical isolates, including 104 carbapenemase producers (97 carbapenemases targeted by the test) and 17 non-carbapenemase producers. The performance of the RESIST ACINETO test was evaluated according to the manufacturer’s recommendations from bacterial and blood cultures. The strains producing the carbapenemases OXA-23, -40, -58, or/and NDM were accurately detected from bacterial cultures and directly from blood cultures, with the exception of one OXA-23/NDM-1-positive *Acinetobacter radioresistens* isolate (only detected through standard culture). None of the non-carbapenemase producers tested positive. The RESIST ACINETO test demonstrated sensitivity/specificity of 100%/100% and 99%/100% on bacterial and blood cultures, respectively.

**IMPORTANCE:**

The incidence of bloodstream infections with carbapenem-resistant *Acinetobacter baumannii* (CRAB) could be very high in some countries such as the Balkans or Southeast Asia. In case of positive blood cultures with Gram-negative bacteria, the use of the RESIST ACINETO test could prove highly beneficial for the rapid identification of these imipenem-resistant bacteria and their antibiotic resistance mechanisms. In addition, it is now well established that New-Delhi Metallo-beta-lactamase (NDM) carbapenemase-producing isolates can have increased MICs of cefiderocol, which is an alternative treatment for these infections. This test may also allow the optimization of treatment based on the type of carbapenemase present. Finally, the RESIST ACINETO test is a rapid, easy-to-use, and cost-effective assay that demonstrates excellent performance in detecting the major acquired carbapenemases present in the *Acinetobacter species*.

## INTRODUCTION

Carbapenem-resistant *Acinetobacter baumannii* (CRAB) poses a significant global threat, leading to a high mortality rate associated with antimicrobial resistance (1). This multidrug- or extensively drug-resistant pathogen is identified by the World Health Organization (WHO) as one of the three critical priority bacteria requiring urgent development of new antibiotics (2). In *Acinetobacter* species, the major mechanism for carbapenem resistance is attributed to the production of carbapenem-hydrolyzing class D beta-lactamases, including OXA-23, OXA-24/40, and OXA-58 groups (3). Additionally, the emerging New-Delhi Metallo-beta-lactamase (NDM) class B carbapenemase plays a significant role in conferring resistance (4, 5). As the activity of the last-line antibiotics, such as the newly developed cefiderocol, depends on the type of carbapenemase, rapid detection of these carbapenemase-producing bacteria has become crucial for optimizing antibiotherapy in the case of an infection and implementing appropriate hygiene measures (6). In recent years, several rapid immunochromatographic tests (Coris BioConcept, NG Biotech) have been developed for the detection of carbapenemase-producing Enterobacterales; but none have been specifically designed for non-fermenting Gram-negative bacilli, with the exception of the Coris OXA-23 K-SeT test (accurate detection of OXA-23 group carbapenemases) (7–9). The novel RESIST ACINETO test (Coris BioConcept) has recently been developed to detect the main carbapenemases identified in the *Acinetobacter* species, namely the OXA-23-, OXA-24/40-, OXA-58-, and NDM-groups (10). Here, we evaluated the performance of this novel immunochromatographic assay for the detection of carbapenemase-producing *Acinetobacter* spp. encountered in mainland and overseas France, from standard and blood cultures.

A large collection of 121 well-characterized *Acinetobacter* spp. clinical strains were selected from the French National Reference Center for Antibiotic Resistance (FNRC-AR) (*n* = 61) and the University Hospital of Reunion Island (*n* = 60). The isolates were identified to the species level using matrix-assisted laser desorption ionization-time of flight (MALDI-TOF) mass spectrometry (MALDI Biotyper; Bruker Daltonics) or by sequencing. The collection included 107 isolates of *A. baumannii calcoaceticus* complex (*A. baumannii n* = 95, *Acinetobacter pittii n* = 10, *Acinetobacter nosocomialis n* = 2), 7 *Acinetobacter ursingii*, 1 *Acinetobacter haemolyticus*, 1 *Acinetobacter johnsonii*, 1 *Acinetobacter junii*, 1 *Acinetobacter proteolyticus*, 1 *Acinetobacter radioresistens*, 1 *Acinetobacter townerii* and 1 *Acinetobacter variabilis*. One hundred and four strains produced 1 (*n* = 75) or 2 carbapenemases (*n* = 29) including 97 enzymes targeted by the test (27 OXA-23 type, 14 OXA-24/40 type, 6 OXA-58 type, 21 NDM-type, 16 OXA-23+NDM-1, 3 OXA-58+NDM-1 and 10 others carbapenemase combinations) (Table S1). Three non-targeted carbapenemases, IMP-63 (*n* = 1) et VIM-4 (*n* = 2), were included as well as four carbapenemase-hydrolyzing class D beta-lactamases (CHDL) rarely identified, OXA-235 (*n* = 1), OXA-255 (*n* = 2) and OXA-679 (*n* = 1). Seventeen non-carbapenemase producers were also included, some of which produced narrow- or broad-spectrum beta-lactamases (Table 1).

**TABLE 1:** Coris ACINETO results obtained with bacterial and blood cultures on the 121 *Acinetobacter* spp. isolates tested^,^^*a*^

Category	Species	Resistance mechanism	Number of strains	Coris ACINETO results obtained with bacterial culture			% Sensitivity (95% CI)	% Specificity (95% CI)	Coris ACINETO results obtained with blood culture			% Sensitivity (95% CI)	% Specificity (95% CI)
				OXA-40/58	OXA-23	NDM			OXA-40/58	OXA-23	NDM		
OXA-23 type (*n* = 27)	*Acinetobacter* spp.	OXA-23	25	N	**P**	N	100 (88–100)	100 (88–100)	N	**P**	N	100 (88–100)	100 (88–100)
	*Acinetobacter* spp.	OXA-565	2	N	**P**	N			N	**P**	N		
OXA-24/40 type (*n* = 14)	*A. baumannii*	OXA-24	5	**P**	N	N	100 (78–100)	100 (78–100)	**P**	N	N	100 (78–100)	100 (78–100)
	*A. baumannii*	OXA-72	9	**P**	N	N			**P**	N	N		
OXA-58 type (*n* = 6)	*A. baumannii*	OXA-58	3	**P**	N	N	100 (61–100)	100 (61–100)	**P**	N	N	100 (61–100)	100 (61–100)
	*Acinetobacter* spp.	OXA-420	3	**P**	N	N			**P**	N	N		
NDM-type (*n* = 21)	*Acinetobacter* spp.	NDM-1	20	N	N	**P**	100 (84–100)	100 (84–100)	N	N	**P**	100 (84–100)	100 (84–100)
	*A. baumannii*	NDM-9	1	N	N	**P**			N	N	**P**		
Multiples carbapenemase producers (*n* = 29)	*A. baumannii*	OXA-23+OXA-58	3	**P**	**P**	N			**P**	**P**	N		
	*A. baumannii*	OXA-23+OXA-420	3	**P**	**P**	N			**P**	**P**	N		
	*A. baumannii*	NDM-1+OXA-23	15	N	**P**	**P**			N	**P**	**P**		
	*A. radioresistens*	NDM-1+OXA-23	1	N	**P**	**P**			N	**N**	**N**		
	*A. baumannii*	NDM-1+OXA-24	1	**P**	N	**P**			**P**	N	**P**		
	*Acinetobacter* spp.	NDM-1+OXA-58	3	**P**	N	**P**			**P**	N	**P**		
	*A. baumannii*	NDM-1+OXA-420	1	**P**	N	**P**			**P**	N	**P**		
	*A. baumannii*	NDM-5+OXA-23	1	N	**P**	**P**			N	**P**	**P**		
	*A. junii*	IMP-37+OXA-58	1	**P**	N	N			**P**	N	N		
Other carbapenemase producers (*n* = 7)	*A. baumannii*	OXA-235	1	N	N	N			N	N	N		
	*A. pittii*	OXA-255	2	**P**	N	N			**P**	N	N		
	*A. pittii*	OXA-679	1	**P**	N	N			**P**	N	N		
	*A. baumannii*	IMP-63	1	N	N	N			N	N	N		
	*Acinetobacter* spp.	VIM-4	2	N	N	N			N	N	N		
Non-carbapenemase producers (*n* = 17)	*A. baumannii*	Wild type	1	N	N	N			N	N	N		
	*A. baumannii*	Overexpressed cephalosporinase	5	N	N	N			N	N	N		
	*A. baumannii*	Overproduction of efflux pumps	2	N	N	N			N	N	N		
	*A. baumannii*	CTX-M-15+CARB-2	1	N	N	N			N	N	N		
	*A. baumannii*	GES-11	1	N	N	N			N	N	N		
	*A. baumannii*	PER-1	1	N	N	N			N	N	N		
	*A. baumannii*	CARB-5	2	N	N	N			N	N	N		
	*A. baumannii*	SHV-12+CTXM-15	1	N	N	N			N	N	N		
	*A. baumannii*	TEM-1	2	N	N	N			N	N	N		
	*A. johnsonii*	Wild type	1	N	N	N			N	N	N		

^*a*^ OXA-255 enzyme has been proposed as a member of the OXA-24/40 subgroup by Yoon and Jeong (11) and could therefore be considered as belonging to this subgroup of CHDL. Dark grey colored boxes correspond to the OXA-23+NDM-1 carbapenemase-producing *A. radioresistens* isolate not detected in blood culture.

^*b*^ Boldface indicates the results obtained with the test and the titles. N, negative; P, positive.

The performance of the RESIST ACINETO test was evaluated according to the manufacturer’s recommendations from standard and blood cultures. From bacterial colonies, a 1 µL full calibrated loop of bacteria grown for 16–24 h at 35 ± 2°C on Mueller*–*Hinton agar plate (bioMérieux) was added to six drops of lysis buffer. After homogenization, 100 µL of the solution was added to the cassette. From blood culture, a blood culture vial (BACTEC Plus Aerobic/F, Becton Dickinson) was inoculated with 1 mL of a bacterial suspension calibrated at 10^2^ CFU/mL (colony forming units) and incubated at 35 ± 2°C in a BACTEC blood culture automated system (Becton Dickinson) until the bacterial inoculum is sufficient to decant the positivity signal. The vial was then removed for processing in <2 h, and 1 mL of the positive blood culture was collected and treated with the RESIST-BC protocol, as recommended by the manufacturer (Fig. S1). Finally, 100 µL of the extraction solution was transferred into the cassette. The reading of cassettes was performed by two operators in a double-blind after 15 min. The sensitivity and specificity of the tests were calculated using the online program of the VassarStats website (http://vassarstats.net).

**FIG 1:**
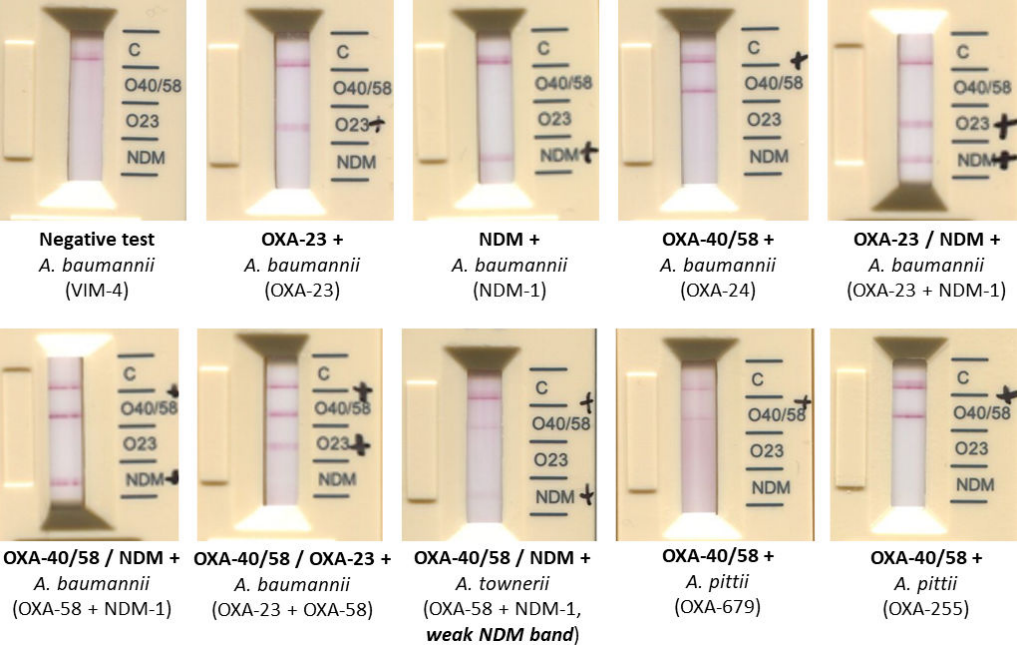
Representation of RESIST ACINETO test results for some *Acinetobacter* spp. isolates of interest.

For the 97 carbapenemase-producing strains targeted by the test, all enzymes were correctly detected from standard and blood cultures, except for the *A. radioresistens* isolate (OXA-23+NDM-1), where both enzymes were only detected from the standard culture (Table 1). However, carbapenem-resistant *A. radioresistens* isolates are very rare, representing <0.1% of *Acinetobacter* species received at the FNRC-AR between 2013 and 2023. The non-detection of this *A. radioresistens* isolate in blood cultures is probably due to an inoculum effect, and also to the low expression of the natural OXA-23-encoding gene, of which this species is the progenitor (12)*.* Seven NDM-producing strains have a low-intensity band particularly from blood culture, with no inter-operator discordance and were considered positive according to the manufacturer’s recommendations (Fig. 1). Interestingly, the test detected in both growth conditions, the strains producing the OXA-255 (*n* = 2) and OXA-679 (*n* = 1) enzymes, which share 87% and 73% amino acid identity with OXA-24/40, respectively (these strains were considered true positives for the performance calculation) (13). Consistent with this result, the OXA-255 enzyme has been proposed as a member of the OXA-24/40 subgroup by Yoon and Jeong (11). However, the test failed to detect the OXA-235 enzyme (OXA-134-like subgroup), which shares an amino acid identity lower than 65% with the enzymes OXA-24/40 (59%), OXA-23 (61%), and OXA-58 (64%) (14). No false positives were observed for the strains producing a non-targeted carbapenemase (IMP-63 and VIM-4) or other beta-lactamases (CTX-M-, GES-, PER-, CARB-, RTG-, TEM-types) (Table 1). The test exhibited a sensitivity of 100% in standard cultures and 99% in blood cultures while demonstrating a specificity of 100% in both conditions. Thus, the RESIST ACINETO test proves to be a reliable method for detecting carbapenemases in *Acinetobacter* species, as demonstrated by three recent studies (10, 15, 16) focusing on standard cultures. In our study, we extended the evaluation to blood culture, revealing consistent results with the previous findings.

These results open up new potential applications for the test based on blood cultures (15, 16). The use of the RESIST ACINETO test could prove highly beneficial for countries with a high incidence of carbapenem-resistant *Acinetobacter* species isolates, particularly in cases where the *A. baumannii-calcoaceticus* complex is responsible for a significant proportion of healthcare-associated bacteremia (e.g., Southeast Asia and the Balkans) (17, 18). When performed directly after the presumptive identification of Gram-negative (coco-)bacilli in a positive blood culture, this test could facilitate the early detection of carbapenem-resistant isolates. It may also allow the optimization of treatment based on the type of carbapenemase present. It is well established that the MICs of cefiderocol against NDM-producing *A. baumannii* isolates are typically higher than 2 mg/L (6). This test could further assist in directing treatment toward alternative therapeutic options (such as colistin, cyclins, or rifampicin) in case of infection.

In conclusion, the RESIST ACINETO test is a rapid, easy-to-use, and cost-effective assay that demonstrates excellent performance in detecting the major acquired carbapenemases present in the *Acinetobacter* species. It has the potential to be considered as an option for routine use in microbiology laboratories.

## SUPPLEMENTAL MATERIAL

Figure S1 spectrum.01044-24-s0001.pdf Blood culture sample processing protocol.

Table S1 spectrum.01044-24-s0002.pdf Molecular characteristics of the 121 *Acinetobacter* spp. isolates included in the study.

ASM does not own the copyrights to Supplemental Material that may be linked to, or accessed through, an article. The authors have granted ASM a non-exclusive, world-wide license to publish the Supplemental Material files. Please contact the corresponding author directly for reuse.
