# Supplementary material for: RESIST ACINETO test for the rapid detection of NDM and OXA acquired carbapenemases directly from blood culture in Acinetobacter species
Source: Microbiol Spectr. 2024 Aug 20;12(10):e01044-24. doi: 10.1128/spectrum.01044-24 (PMC11448386; doi:10.1128/spectrum.01044-24)
Supplement: Figure S1 — Blood culture sample processing protocol. [file spectrum.01044-24-s0001.pdf]

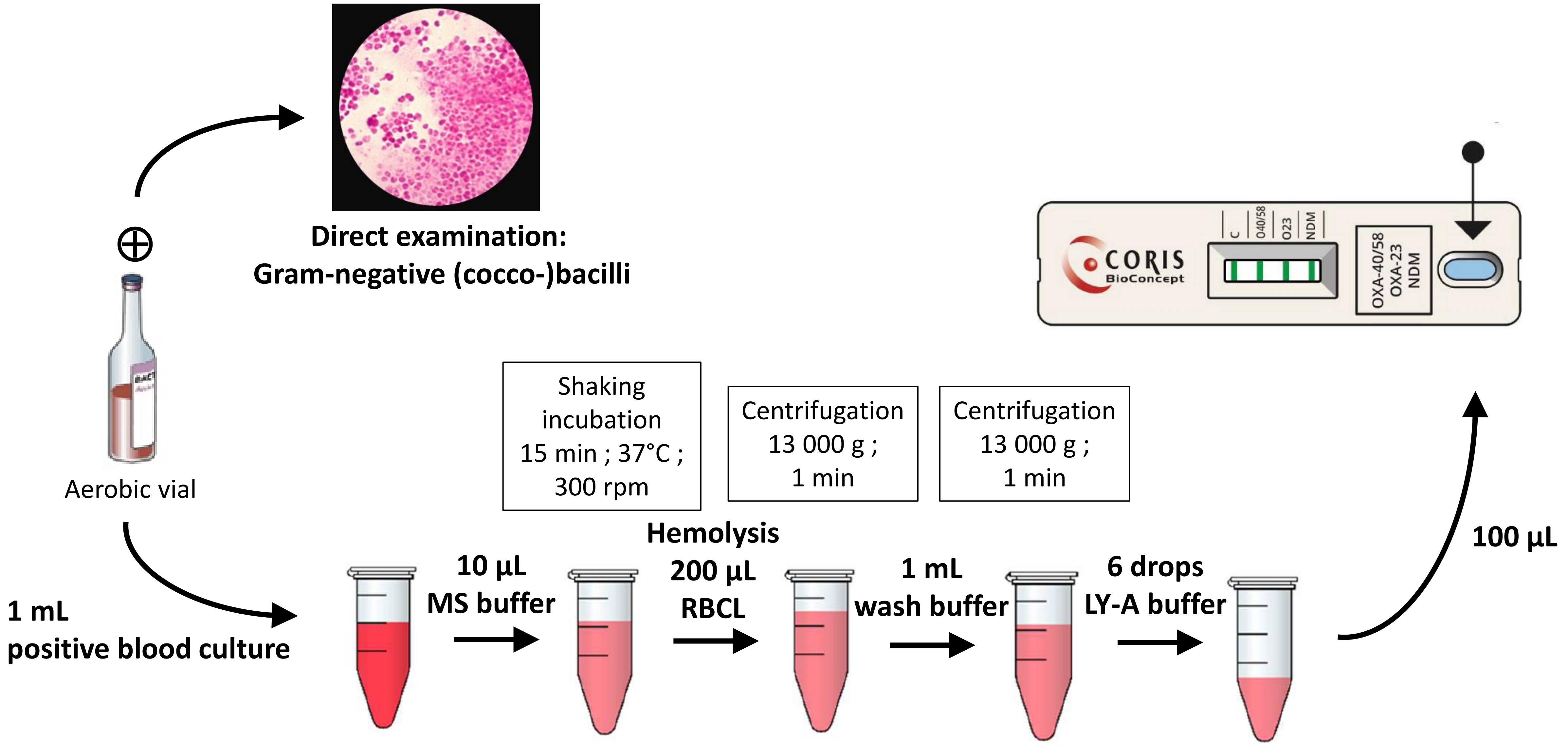

Figure S1: Blood culture sample processing protocol (adapted from RESIST-BC protocol, Coris BioConcept, technical note n°IFU-57S01/FR/V02, https://www.corisbio.com/products/resist-acineto)
