# Supplementary material for: RESIST ACINETO test for the rapid detection of NDM and OXA acquired carbapenemases directly from blood culture in Acinetobacter species
Source: Microbiol Spectr. 2024 Aug 20;12(10):e01044-24. doi: 10.1128/spectrum.01044-24 (PMC11448386; doi:10.1128/spectrum.01044-24)
Supplement: Table S1 — Molecular characteristics of the 121 Acinetobacter spp. isolates included in the study. [file spectrum.01044-24-s0002.pdf]

Table S1. Molecular characteristics of the 121 *Acinetobacter* spp. isolates included in the study.

	Strain number	Species	Strain origin	Carbapenemases		
				Class B	Class D	Other beta-lactamase(s)
**1**	13A.297	*Acinetobacter baumannii*	France	NDM-9		
**2**	14A.630	*Acinetobacter baumannii*	France			PER-1
**3**	14A.728	*Acinetobacter baumannii*	France		OXA-72	
**4**	15A.838	*Acinetobacter baumannii*	India	NDM-1	OXA-23	PER-7
**5**	15A.1119	*Acinetobacter baumannii*	France[¤]		OXA-72	CTX-M-115, TEM-1
**6**	16A.1365	*Acinetobacter baumannii*	Morocco		OXA-23, -58	
**7**	16A.1546	*Acinetobacter pittii*	France		OXA-255	
**8**	16A.1547	*Acinetobacter baumannii*	France			RTG-2 (CARB-5)
**9**	17A.1578	*Acinetobacter baumannii*	France	NDM-1	OXA-24	
**10**	17A.1728	*Acinetobacter baumannii*	Spain		OXA-24	TEM-1
**11**	17A.1866	*Acinetobacter baumannii*	Senegal			TEM-1
**12**	17A.1882	*Acinetobacter baumannii*	Morocco	NDM-1	OXA-23	
**13**	17A.1911	*Acinetobacter baumannii*	Georgia		OXA-72	GES-22
**14**	17A.1934	*Acinetobacter pittii*	France	VIM-4		
**15**	17A.1955	*Acinetobacter baumannii*	France	NDM-1		
**16**	17A.1988	*Acinetobacter haemolyticus*	France	NDM-1		
**17**	18A.2067	*Acinetobacter baumannii*	France		OXA-72	
**18**	18A.2089	*Acinetobacter baumannii*	Ethiopia			GES-11
**19**	18A.2101	*Acinetobacter junii*	France	IMP-37	OXA-58	TEM-2, OXA-35
**20**	19A.2631	*Acinetobacter baumannii*	Cambodia		OXA-23	CARB-16
**21**	18A.2288	*Acinetobacter baumannii*	France		OXA-420	
**22**	18A.2354	*Acinetobacter baumannii*	France		OXA-58	PER-13
**23**	19A.2514	*Acinetobacter baumannii*	Tchad	NDM-1	OXA-58	
**24**	19A.2560	*Acinetobacter baumannii*	France	NDM-1		
**25**	19A.2708	*Acinetobacter baumannii*	France		OXA-72	
**26**	19A.2732	*Acinetobacter baumannii*	France[♦]			
**27**	19A.2749	*Acinetobacter baumannii*	France			
**28**	19A.2755	*Acinetobacter baumannii*	Tunisia	NDM-1	OXA-23	TEM-1
**29**	19A.2766	*Acinetobacter baumannii*	France	NDM-1	OXA-23	TEM-1
**30**	19A.2809	*Acinetobacter baumannii*	Senegal		OXA-23, -420	
**31**	19A.2835	*Acinetobacter ursingii*	Lebanon	VIM-4		
**32**	19A.2838	*Acinetobacter baumannii*	Hongria		OXA-23, -58	
**33**	20A.2880	*Acinetobacter baumannii*	Gabon		OXA-23, -420	
**34**	20A.2970	*Acinetobacter baumannii*	France			
**35**	20A.2979	*Acinetobacter baumannii*	Equatorial Guinea			TEM-1
**36**	20A.2993	*Acinetobacter pittii*	France		OXA-255	
**37**	20A.2994	*Acinetobacter baumannii*	Algeria		OXA-24	
**38**	20A.3025	*Acinetobacter baumannii*	France			
**39**	20A.3068	*Acinetobacter baumannii*	France			
**40**	21A.3076	*Acinetobacter baumannii*	Morocco		OXA-23	GES-11
**41**	21A.3085	*Acinetobacter baumannii*	Senegal		OXA-23, -420	
**42**	21A.3102	*Acinetobacter baumannii*	Senegal		OXA-23, -58	
**43**	21A.3125	*Acinetobacter baumannii*	Turkey			
**44**	21A.3147	*Acinetobacter baumannii*	France	NDM-1		
**45**	21A.3148	*Acinetobacter baumannii*	France	NDM-1	OXA-420	
**46**	21A.3224	*Acinetobacter baumannii*	France		OXA-72	PER-1
**47**	21A.3226	*Acinetobacter baumannii*	Congo	NDM-1	OXA-58	CARB-16
**48**	21A.3238	*Acinetobacter baumannii*	France			CTX-M-15, PSE-1
**49**	21A.3253	*Acinetobacter baumannii*	France	NDM-1		PER-7
**50**	21A.3262PER	*Acinetobacter baumannii*	Ouzbekistan		OXA-72	PER-1
**51**	21A.3272	*Acinetobacter baumannii*	France	IMP-63		OXA-35
**52**	21A.3273	*Acinetobacter baumannii*	France		OXA-235	
**53**	22A.3312	*Acinetobacter baumannii*	France		OXA-72	
**54**	22A.3333	*Acinetobacter baumannii*	Ivory Coast		OXA-420	
**55**	22A.3373	*Acinetobacter baumannii*	France			
**56**	22A.3391	*Acinetobacter johnsonii*	France			
**57**	16A.1487	*Acinetobacter baumannii*	France		OXA-565	
**58**	16A.1504	*Acinetobacter pittii*	France		OXA-679	
**59**	22A.3404	*Acinetobacter baumannii*	France	NDM-5	OXA-23	
**60**	15A.1113	*Acinetobacter ursingii*	France[#]	NDM-1		
**61**	15A.1167	*Acinetobacter townerii*	Comoros	NDM-1	OXA-58	
**62**	19A.2764	*Acinetobacter baumannii*	Madagascar		OXA-23	
**63**	15A.1108	*Acinetobacter baumannii*	Mauritius		OXA-23	
**64**	18A.2278	*Acinetobacter baumannii*	France		OXA-24	
**65**	14A.776	*Acinetobacter baumannii*	Comoros		OXA-58	
**66**	19A.2680	*Acinetobacter baumannii*	France*	NDM-1		
**67**	19A.2470	*Acinetobacter pittii*	France*	NDM-1		
**68**	20A.266	*Acinetobacter ursingii*	France*	NDM-1		
**69**	20A.2977	*Acinetobacter baumannii*	France*	NDM-1	OXA-23	
**70**	16A.1260	*Acinetobacter radioresistens*	Comoros	NDM-1	OXA-23	
**71**	19A.2511	*Acinetobacter pittii*	France*	NDM-1		
**72**	17A.1684	*Acinetobacter baumannii*	Madagascar		OXA-23	
**73**	22A.3322	*Acinetobacter baumannii*	France*		OXA-23	
**74**	17A.1618	*Acinetobacter baumannii*	Madagascar		OXA-23	TEM-1
**75**	18A.2171	*Acinetobacter baumannii*	Madagascar		OXA-23	TEM-1
**76**	17A.2036	*Acinetobacter baumannii*	Madagascar		OXA-23	
**77**	22A.3545	*Acinetobacter baumannii*	France[#]		OXA-23	
**78**	18A.2283	*Acinetobacter baumannii*	France*		OXA-23	TEM-1
**79**	17A.1631	*Acinetobacter baumannii*	France*		OXA-23	TEM-1
**80**	22A.3306	*Acinetobacter baumannii*	France*		OXA-23	
**81**	18A.2172	*Acinetobacter baumannii*	France*		OXA-23	
**82**	19A.2790	*Acinetobacter baumannii*	France*		OXA-23	
**83**	20A.2966	*Acinetobacter baumannii*	France*		OXA-23	
**84**	19A.2607	*Acinetobacter baumannii*	France*		OXA-23	
**85**	18A.2205	*Acinetobacter baumannii*	France*		OXA-23	AmpC, ArmA
**86**	22A.286	*Acinetobacter baumannii*	France*		OXA-23	
**87**	22A.3381	*Acinetobacter baumannii*	France		OXA-23	TEM-1
**88**	17A.2007	*Acinetobacter baumannii*	France*		OXA-23	TEM-1
**89**	16A.1437	*Acinetobacter baumannii*	France*		OXA-23	TEM-1
**90**	16A.1426	*Acinetobacter baumannii*	France*		OXA-23	
**91**	16A.1319	*Acinetobacter baumannii*	Mauritius		OXA-23	
**92**	16A.1378	*Acinetobacter baumannii*	France*	NDM-1	OXA-23	
**93**	19A.2547	*Acinetobacter baumannii*	France*		OXA-24	
**94**	22A.3546	*Acinetobacter baumannii*	Madagascar		OXA-24	
**95**	15A.919	*Acinetobacter baumannii*	Ivory Coast		OXA-58	
**96**	22A.3374	*Acinetobacter nosocomialis*	France[#]		OXA-420	
**97**	21A.3114	*Acinetobacter baumannii*	France*		OXA-72	
**98**	17A.917	*Acinetobacter baumannii*	France[#]	NDM-1		
**99**	21A.3294	*Acinetobacter proteolyticus*	France[#]	NDM-1		
**100**	22A.3317	*Acinetobacter ursingii*	France[#]	NDM-1		
**101**	21A.3157	*Acinetobacter ursingii*	France[#]	NDM-1		
**102**	21A.3129	*Acinetobacter variabilis*	France[#]		OXA-565	
**103**	16A.784	*Acinetobacter baumannii*	France[#]	NDM-1	OXA-23	
**104**	20A.3064	*Acinetobacter pittii*	France*	NDM-1		
**105**	22A.3430	*Acinetobacter ursingii*	France*	NDM-1		
**106**	20A.2915	*Acinetobacter nosocomialis*	France*	NDM-1		
**107**	17A.1971	*Acinetobacter pittii*	France*	NDM-1		
**108**	17A.2022	*Acinetobacter ursingii*	France*	NDM-1		
**109**	21A.3138	*Acinetobacter baumannii*	France[#]	NDM-1	OXA-23	
**110**	19A.2575	*Acinetobacter baumannii*	France*	NDM-1	OXA-23	
**111**	23A.3654	*Acinetobacter baumannii*	France*	NDM-1	OXA-23	
**112**	22A.3316	*Acinetobacter baumannii*	France*	NDM-1	OXA-23	
**113**	18A.2363	*Acinetobacter baumannii*	France*	NDM-1	OXA-23	
**114**	20A.2972	*Acinetobacter baumannii*	France*	NDM-1	OXA-23	
**115**	14A.756	*Acinetobacter baumannii*	Madagascar		OXA-23	PER-7
**116**	18A.2409	*Acinetobacter baumannii*	France*	NDM-1	OXA-23	
**117**	18A.2116	*Acinetobacter pittii*	France[#]	NDM-1		
**118**	23A.2298	*Acinetobacter baumannii*	France	NDM-1	OXA-23	
**119**	19A.2859	*Acinetobacter baumannii*	France*			RTG-2 (CARB-5)
**120**	14A.607	*Acinetobacter baumannii*	France			SHV-12 + CTX-M-15
**121**	15A.695	*Acinetobacter baumannii*	France[#]			

[¤] Guadeloupe
[♦] Guyane
[#] Mayotte
* Reunion
